# Effectiveness of strength-oriented rehabilitation interventions as a non-pharmacologic rehabilitation strategy for knee function after anterior cruciate ligament reconstruction: a three-level meta-analysis

**DOI:** 10.3389/fmed.2026.1871508

**Published:** 2026-06-24

**Authors:** Rui Wang, Guang Cao, Hongliang Wang, Shihua Guo, Shiwei Chen, Xue Han

**Affiliations:** 1 College of Physical Education and Health Management, Henan Finance University, Zhengzhou, China; 2 School of Physical Education and Health, Yancheng Normal University, Yancheng, China; 3 School of Physical Education, Zhengzhou University of Science and Technology, Zhengzhou, China; 4 School of Sport Science, Beijing Sport University, Beijing, China; 5 Rehabilitation Center, Hebei Institute of Sports Science, Shijiazhuang, China; 6 Key Laboratory of Training Load Diagnosis and Regulation for Elite Athletes, General Administration of Sport of China, Shijiazhuang, China; 7 Hebei Key Laboratory of Digital Physical Fitness Monitoring and Health Promotion, Shijiazhuang, China

**Keywords:** ACL reconstruction, hop performance, knee strength, strength training, three-level meta-analysis

## Abstract

**Background:**

Persistent deficits in periarticular muscle strength and functional performance are commonly observed following anterior cruciate ligament reconstruction, potentially compromising long-term knee joint health. As a key non-pharmacologic rehabilitation strategy, strengthening exercise is widely implemented to address these impairments. However, evidence regarding its effects on knee muscle strength and hop-performance outcomes remains inconsistent. Therefore, this study aimed to systematically quantify the effects of strengthening exercise on knee muscle strength and hop performance after ACLR using a three-level meta-analytic approach.

**Methods:**

PubMed, Web of Science, Embase, Cochrane Library, and SPORTDiscus were searched through January 6, 2026. Randomized controlled trials examining strengthening-based rehabilitation after ACLR were included. Standardized mean differences (Hedges’ g) were calculated from pre–post change scores. Three-level multivariate random-effects meta-analyses were conducted, with moderator analyses examining postoperative time, population type, and assessment time point. Risk of bias was assessed using RoB 2, and publication bias was explored via funnel plots and Egger’s regression.

**Results:**

Nine trials were included, contributing a total of 24 effect sizes across outcomes. Strengthening exercise significantly improved knee muscle strength (*d* = 0.39, 95% CI 0.01–0.77, *p* = 0.046, *I*^2^ = 55.8%). No significant effect was found for hop performance (*d* = −0.03, 95% CI − 0.54 to 0.48, *p* = 0.906, *I*^2^ = 77.8%). No statistically significant moderation effects of postoperative time, population type, or assessment time point were detected for either outcome.

**Conclusion:**

Strength-oriented rehabilitation interventions appear to improve knee muscle strength following ACL reconstruction, whereas no significant overall effect was observed for hop performance. Comprehensive rehabilitation strategies addressing neuromuscular and functional demands may be required to optimize postoperative recovery.

## 1 Introduction

Anterior cruciate ligament (ACL) injury is one of the most common and burdensome sports-related knee injuries, particularly in sports involving cutting, pivoting, jump landings, and physical contact (1). Because ACL rupture may result in knee instability, functional limitations, and an increased risk of subsequent injury, anterior cruciate ligament reconstruction (ACLR) has been widely performed to restore mechanical stability and facilitate return to physical activity (2). However, improvements in “structural stability” do not necessarily translate into full functional recovery. Many patients continue to experience challenges in return to sport (RTS) and returning to preinjury levels, and returning to competitive sport, suggesting a persistent gap in real-world functional outcomes after ACLR (3).

Among the determinants of long-term functional limitations after ACLR, deficits in periarticular muscle strength are considered one of the most critical and prevalent impairments. Recent systematic reviews indicate that many patients exhibit persistent and long-lasting deficits in quadriceps and hamstring strength after ACLR (4). Mechanistically, arthrogenic muscle inhibition (AMI) has been implicated in impaired muscle activation following ACL injury and reconstruction. Scoping reviews have summarized evidence that AMI may persist throughout rehabilitation and may adversely affect quadriceps activation and overall functional recovery (5, 6). Therefore, restoring knee extensor and flexor strength as well as neuromuscular activation remains a central goal and challenge in postoperative rehabilitation.

In addition to strength deficits, impaired hopping functional performance are also common after ACLR and directly influence RTS assessment and decision-making. Hop tests have been linked not only to current function but also to the prediction of future symptoms, function, and RTS outcomes (7). Longitudinal evidence syntheses suggest that hop performance generally improves over time after surgery, yet the recovery trajectory does not always equate to achieving an “ideal” functional level (8).

At the same time, systematic reviews of RTS test batteries have emphasized that reliance on a single category of metrics (e.g., hop distance alone) may be insufficient to identify residual impairments, supporting the need for a more comprehensive evaluation incorporating strength, hop performance, and other relevant domains (9).

Strengthening exercise, with a particular emphasis on the quadriceps and hamstring muscle groups, represents a core component of rehabilitation following ACL reconstruction, as it plays a critical role in modulating joint loading mechanics, graft stress, and neuromuscular control during movement (10). Functionally, the quadriceps and hamstrings serve as key dynamic stabilisers of the knee, helping to resist anterior tibial translation and rotational forces acting on the reconstructed ligament, thereby potentially minimising excessive strain on the graft (11).

From a neuromechanical standpoint, progressive resistance training may facilitate improved motor unit activation, attenuate postoperative muscle wasting, and enhance proprioceptive feedback and motor coordination (12). Collectively, these adaptations contribute to more effective functional recovery and improved performance outcomes after ACLR (13).

Despite a growing body of research examining the effects of strengthening exercise after ACL reconstruction, existing findings remain fragmented and sometimes inconsistent (14). Conventional meta-analyses rely on the assumption of independent effect sizes, typically extracting a single effect size from each study (15). However, research on strengthening interventions following ACLR frequently reports multiple effect sizes derived from the same sample, such as outcomes for different strength assessment modes (e.g., isometric peak torque, eccentric peak torque), and multiple assessment time points (e.g., post-intervention and follow-up) (16, 17). Restricting meta-analyses to a single effect size per study may therefore result in substantial loss of information and limit the ability to fully capture the complexity and variability of the effects of strengthening exercise on postoperative recovery.

To address these methodological limitations, the present study employs a three-level meta-analysis approach, which allows for the inclusion of all relevant effect sizes while appropriately accounting for their statistical dependence (18). By simultaneously modeling sampling variance (Level 1), within-study variance among multiple effect sizes (Level 2), and between-study variance (Level 3), this approach enables a more precise and comprehensive synthesis of the existing evidence (19). Through this comprehensive synthesis, the present study aims to clarify the overall effects of strengthening exercise on knee muscle strength and hop performance following ACL reconstruction.

## 2 Methods

This study followed established PRISMA reporting standards for systematic reviews and meta-analyses (20). The PRISMA checklist is provided in the Supplementary materials. The protocol was registered on PROSPERO (CRD420261309251).

### 2.1 Literature search and selection

A systematic literature search was conducted to identify studies investigating the effects of strengthening exercise interventions on knee muscle strength and hop performance following anterior cruciate ligament reconstruction. Searches were performed across the following electronic databases: PubMed, Web of Science, Embase, Cochrane Library, and SPORTDiscus (via EBSCOhost). All eligible records published up to January 6, 2026, were considered.

The search strategy was structured around three core concept domains: (1) ACL reconstruction–related populations, (2) strengthening or resistance-based exercise interventions, and (3) outcomes related to muscle strength and hop performance. Specifically, search terms included variations of ACL reconstruction and surgery (e.g., “anterior cruciate ligament reconstruction,”“ACL reconstruction,” ACLR, “ACL,” “ACL surgery”), combined with terms describing strengthening interventions (e.g., strength training, resistance training, eccentric training, concentric training, isometric training, neuromuscular training, quadriceps strengthening), as well as outcome-related terms capturing muscle strength and hop or jump performance (e.g., muscle strength, power, performance, function, hop tests, single-leg hop, jumping performance).

All three search components were combined using Boolean operators to ensure that retrieved studies simultaneously addressed the target population, intervention type, and relevant outcomes. Searches were applied to titles, abstracts, and keywords. The complete search strategy is provided in Supplementary File 1.

Additionally, no language restrictions were applied during the literature search. Trial registries and grey literature sources were also searched to identify potentially eligible studies. When required data were unavailable or incomplete, attempts were made to contact the corresponding authors of the included studies to obtain additional information.

### 2.2 Inclusion and exclusion criteria

Studies were considered eligible if they met the following criteria: (1) participants were individuals who had undergone anterior cruciate ligament reconstruction; (2) the intervention involved strengthening exercise–based rehabilitation programs, including resistance training, eccentric or concentric strengthening, isometric exercise, or neuromuscular strengthening protocols; (3) the study included a comparison group receiving usual care, standard rehabilitation, alternative rehabilitation approaches, or alternative strength-oriented rehabilitation interventions. (4) outcomes reported measures of knee muscle strength (e.g., peak torque) and/or functional hop performance (e.g., single-leg hop tests); and (5) the study design was a randomized controlled trial. In addition, sufficient statistical information had to be available to calculate standardized effect sizes, including means and standard deviations for pre- and post-intervention assessments for both intervention and comparison groups.

Studies were excluded if they: (1) were not randomized controlled trials (e.g., observational studies, quasi-experimental designs, case series); (2) did not involve postoperative ACLR populations; (3) lacked a strengthening-focused intervention; (4) did not report relevant strength or hop-related outcomes; or (5) were conference abstracts, reviews, protocols, or non-peer-reviewed publications. Insufficient statistical data were reported to compute standardized effect sizes, such as the absence of means and standard deviations for pre- and post-intervention assessments in either the intervention or comparison groups.

Participants were classified as athletes when the original study explicitly described them as competitive, elite, professional, collegiate, or regularly participating athletes. Participants were classified as general patients when no specific athletic status was reported and the sample was recruited from the general ACL reconstruction population.

### 2.3 Data extraction and coding process

Data were independently extracted from all included randomized controlled trials using a standardized data extraction form. The following information was collected: first author and year of publication, country, total sample size, population characteristics (e.g., athletes or general patients), intervention type, intervention duration and frequency, outcome measures, and baseline body mass index (BMI) where available.

Strength-oriented rehabilitation interventions were defined as exercise programs primarily intended to improve lower-limb muscle strength, force-generating capacity, or strength-related functional performance following ACL reconstruction. These interventions included progressive resistance training, eccentric strengthening, isokinetic strengthening, and neuromuscular training programs with a primary strength-development component.

Outcome data included measures of knee muscle strength (e.g., peak torque, maximal isometric strength, concentric or eccentric strength) and hop performance outcomes (e.g., single-leg hop tests). Strength outcomes included measures of quadriceps and hamstring strength assessed using isometric, concentric, and eccentric testing modalities. Although these measures represent different aspects of muscle performance, they were considered indicators of knee muscle strength and were pooled to evaluate overall muscle force-generating capacity around the knee joint following ACL reconstruction. For each eligible outcome, means and standard deviations were extracted for both intervention and comparison groups at pre- and post-intervention time points to enable the calculation of standardized effect sizes.

When multiple outcomes or time points were reported within a single study, each relevant effect size was coded separately while accounting for within-study dependency in the multilevel meta-analytic model. Missing or unclear information (e.g., BMI or intervention frequency) was recorded as not available.

All data extraction and coding procedures were conducted independently by two pairs of reviewers (R. W. and G. C.; H. W. and S. G.). Extracted data were cross-checked for accuracy, and any discrepancies were resolved through discussion and adjudication by the corresponding author (S. C. and X. H.).

### 2.4 Risk of Bias and publication Bias assessment and certainty of evidence

The risk of bias in the included randomized controlled trials was independently assessed by two pairs of reviewers (R. W. and G. C.; H. W. and S. G.) using the Cochrane Risk of Bias tool (RoB 2), which evaluates five domains: the randomization process, deviations from intended interventions, missing outcome data, outcome measurement, and selection of the reported result (21). Any disagreements were resolved through discussion or consultation with a third reviewer (S. C. and X. H.).

Potential publication bias and small-study effects were evaluated through visual inspection of funnel plots and Egger’s regression test (22). For this purpose, a conventional two-level random-effects model was fitted to obtain effect size estimates and corresponding standard errors. Funnel plots were constructed by plotting effect sizes against their standard errors, with pseudo 95% confidence intervals to assist interpretation.

Egger’s regression test was performed using a mixed-effects meta-regression model with the standard error entered as the predictor. A statistically significant regression coefficient was considered indicative of potential funnel plot asymmetry (23). Given the relatively small number of effect sizes and the dependency structure inherent in the three-level meta-analytic framework, findings were interpreted cautiously.

The certainty of evidence for knee muscle strength and hop performance outcomes was evaluated using the GRADE framework (24). Because all included studies were randomized controlled trials, the evidence initially started at high certainty and was downgraded when warranted across the domains of risk of bias, inconsistency, indirectness, imprecision, and publication bias.

### 2.5 Statistical analysis

All meta-analyses were conducted using multilevel random-effects models to account for the dependency of multiple effect sizes extracted from the same study (15). Standardized mean differences (SMDs, Cohen’s d) were calculated using post-intervention means and standard deviations from the intervention and comparison groups. The pooled standard deviation (SDpooled) was calculated using Equation (1):


$$SD_{\text{pooled}}=\sqrt{\frac{(n_{\text{post}\_\exp}-1)\times SD_{\text{post}\_\exp}^{2}+(n_{\text{post}\_\text{ctrl}}-1)\times SD_{\text{post}\_\text{ctrl}}^{2}}{n_{\text{post}\_\exp}+n_{\text{post}\_\text{ctrl}}-2}}$$ (1)

The standardized mean difference for each study was then calculated using Equation (2):


$$d_{\exp\_\text{ctrl}}=\frac{M_{\text{post}\_\exp}-M_{\text{post}\_\text{ctrl}}}{SD_{\text{pooled}}}$$ (2)

The sampling variance of the SMD was calculated using Equation (3):


$$\mathrm{Va}r_{d_{\exp\_\text{ctrl}}}=\frac{n_{\text{post}\_\exp}+n_{\text{post}\_\text{ctrl}}}{n_{\text{post}\_\exp}\times n_{\text{post}\_\text{ctrl}}}+\frac{{d^{2}}_{\exp\_\text{ctrl}}}{2\times (n_{\text{post}\_\exp}+n_{\text{post}\_\text{ctrl}})}$$ (3)

Effect sizes from the same study that were statistically dependent (e.g., multiple outcomes) were handled using a three-level random-effects meta-analysis model (`rma.mv` function in the R metafor package), with effect sizes nested within studies. Restricted maximum likelihood estimation (REML) was used to estimate model parameters.

Statistical heterogeneity was assessed using the Q statistic and quantified with the *I*^2^ index (25). Variance components were further decomposed to estimate the proportion of heterogeneity attributable to within-study (Level 2) and between-study (Level 3) variability. Moderator analyses were conducted using mixed-effects meta-regression to examine potential influences of clinical and methodological factors, including postoperative time point and population type.

All analyses were conducted in R (version 4.3.2; (39)) using the metafor package (26). Statistical significance was set at *p* < 0.05, and results were presented using forest plots and additional graphical displays where applicable.

## 3 Results

### 3.1 Search results and study characteristics

The literature search covered studies published up to January 6, 2026. A total of 1,671 records were retrieved from electronic databases, including PubMed, Web of Science, Embase, Cochrane Library, and SPORTDiscus. In addition, five records were identified through alternative sources such as website searches and citation tracking.

**Figure 1:**
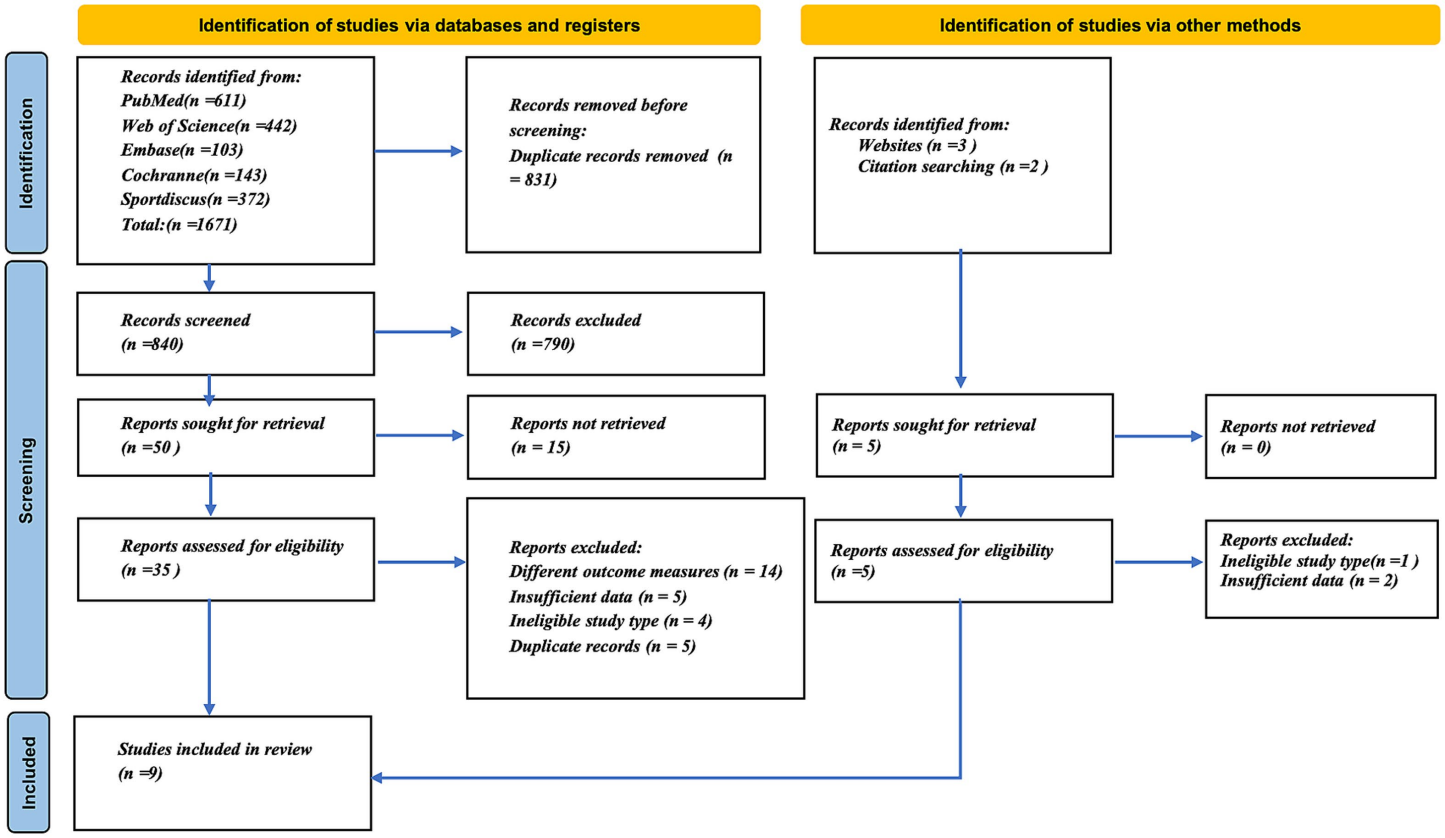
PRISMA flow diagram of study selection.

Following the removal of 831 duplicate records, 840 records remained for title and abstract screening. Of these, 790 records were excluded. Fifty reports were subsequently sought for full-text retrieval, of which 15 could not be obtained. Consequently, 35 reports from database searches were assessed for eligibility. After full-text evaluation, 28 reports were excluded for the following reasons: different outcome measures (*n* = 14), insufficient data (*n* = 5), ineligible study design (*n* = 4), and duplicate records (*n* = 5). From other sources, five reports were sought for retrieval, all of which were successfully obtained and assessed for eligibility. Three reports were subsequently excluded due to ineligible study design (*n* = 1) or insufficient data (*n* = 2). Ultimately, nine studies (16, 17, 27–33) met the predefined inclusion criteria and were included in the final review. The detailed study selection process is presented in Figure 1.

The studies included in this review were carried out in diverse geographical settings, encompassing Turkey, Denmark, Brazil, the United States, India, Serbia, Pakistan, Spain, and Australia. Across trials, sample sizes ranged from 25 to 103 participants, involving both general patient populations and athletic groups. Outcome assessment primarily focused on knee flexor and extensor strength as well as hop performance. Strength-related measures included peak torque during knee flexion and extension, assessed under isometric, concentric, and eccentric contraction modes, while hop performance was frequently evaluated using the single-leg hop test.

Intervention strategies were predominantly strength-oriented and varied across studies. These protocols incorporated progressive strength training, eccentric resistance exercise, neuromuscular strengthening, as well as isokinetic eccentric and isoinertial training approaches. Training programs were delivered at frequencies of 2 to 6 sessions per week, with intervention durations spanning from 5 to 12 weeks. Baseline BMI values were generally reported within the normal range. A detailed overview of study characteristics is provided in Table 1.

**Table 1:** Characteristics of included literature.

Author	Country	N (total sample)	Measure	Population	Intervention	Duration and frequency	BMI (kg/m^2^)
Kınıklı et al. (2014) (16)	Turkey	33	Peak torque of knee flexion Single-leg hop	General patients	Progressive strength training	3 times a week for 12 weeks	E:24.50±2.36 C:24.52±0.94
Bregenhof et al. (2023) (27)	Denmark	51	Maximal isometric knee flexor strength	Athletes	Progressive strength training	2 times a week for 12 weeks	E:25.6 ±4.5 C:24.5 ±3.4
Vidmar et al. (17)	Brazil	30	Isometric peak torque Concentric peak torque Eccentric peak torque	Athletes	Isokinetic eccentric training program	2 times a week for 6 weeks	E:23.4 ± 1.0 C:23.7 ± 1.2
Gerber et al. (28)	USA	40	Quadriceps strength Single-leg hop	General patients	Eccentric resistance training	2 times a week for 12 weeks	25.27 ± 5.49
Patra et al. (29)	India	96	Isometric peak torque	General patients	Isoinertial training	6 times a week for 6 weeks	26.57 ±3.81
Stojanović et al. (30)	Serbia	44	Isometric peak torque Single-leg hop	Athletes	Isokinetic eccentric training	3 times a week for 6 weeks	23.3 ± 5.6
Khalid et al. (31)	Pakistan	76	Single-leg hop	General patients	Neuromuscular strength training	3 times a week for 6 weeks	N/A
Jiménez-Rubio et al. (32)	Spain	25	Single-leg hop	Athletes	Progressive strength training	3 times a week for 12 weeks	21.85 ± 0.7
Shaw et al. (33)	Australia	103	Single-leg hop	General patients	Progressive strength training	N/A	25.3±4.2

### 3.2 Risk of bias and publication bias and certainty of evidence

**Figure 2:**
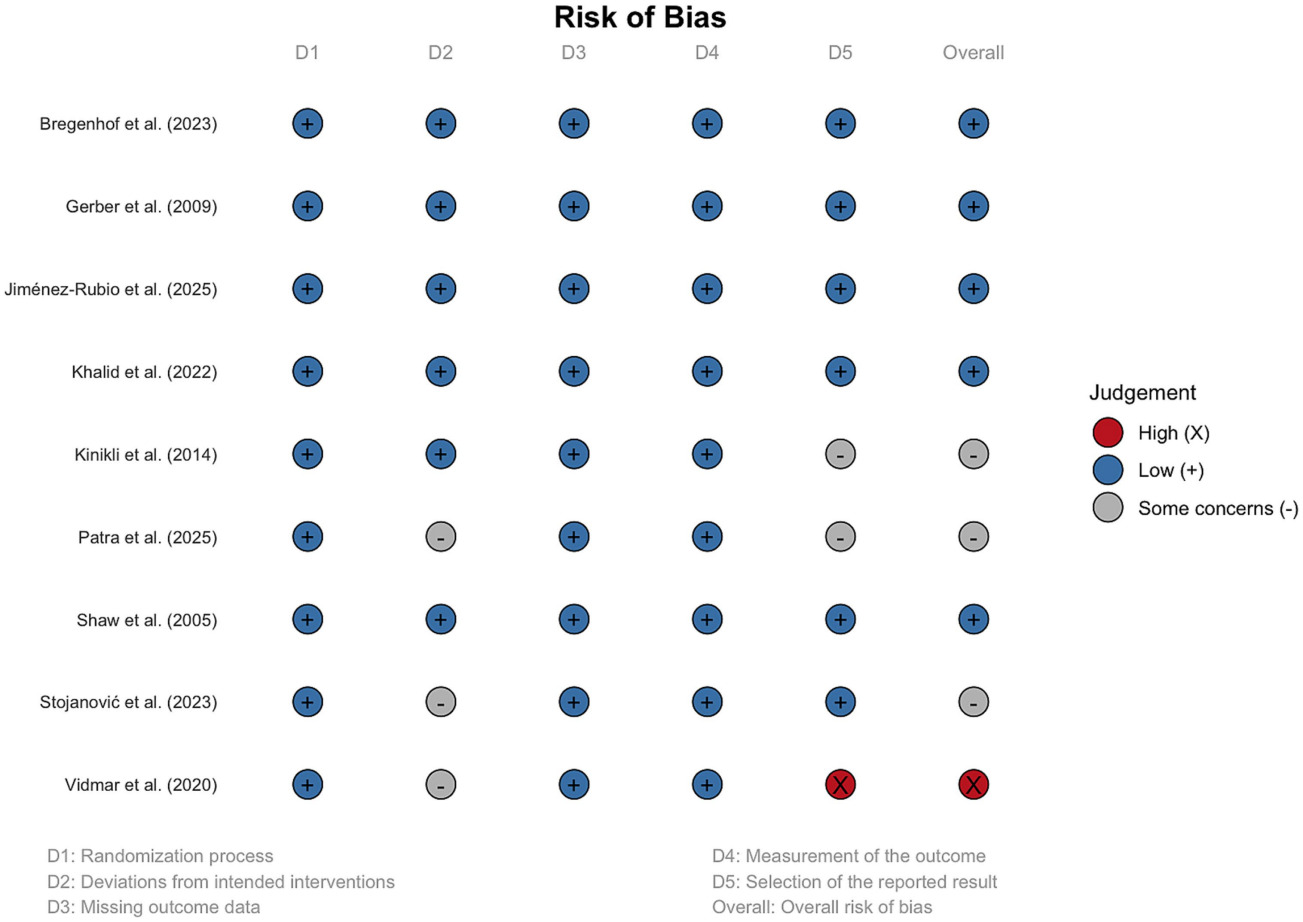
Risk of bias assessment based on the ROB-2 tool.

Risk of bias was evaluated using the Cochrane RoB 2 tool. Overall, the methodological quality of the included trials was generally acceptable. Five studies were assessed as having a low risk of bias, three were judged to raise some concerns, and one study was rated as high risk of bias. Most domains showed low risk across studies, particularly for the randomization process, deviations from intended interventions, and outcome measurement. The main sources of uncertainty were related to missing outcome data and potential selective reporting. Detailed domain-level assessments are provided in Figure 2.

**Figure 3:**
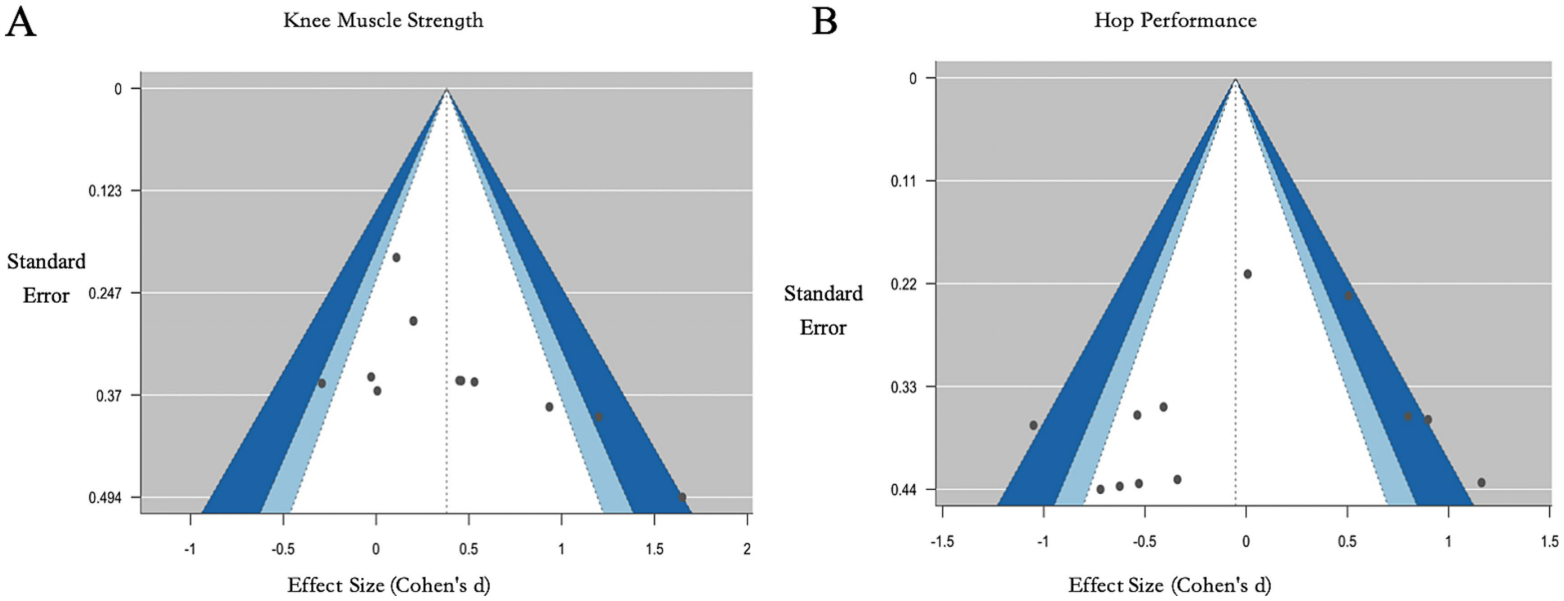
Funnel plots for publication bias assessment. **(A)** Knee muscle strength. **(B)** Hop performance. The vertical dashed line indicates the pooled effect estimate. The white triangular region represents the 95% pseudo-confidence limits. Points represent individual effect sizes.

Funnel plot asymmetry was assessed for both knee muscle strength and hop performance outcomes using mixed-effects meta-regression models with the standard error as the predictor. For knee muscle strength, the regression test suggested statistically significant asymmetry (z = 2.48, *p* = 0.013). However, given the limited number of effect sizes and the dependency structure of the data, this finding should be considered exploratory and interpreted with caution. The estimated limit effect size as the standard error approached zero was b = −0.93 (95% CI: −1.98 to 0.11), with the confidence interval including zero. Detailed results are presented in Figure 3A.

In contrast, for hop performance, no significant funnel plot asymmetry was detected (z = −0.79, *p* = 0.429). However, the small number of included effect sizes limits the ability to reliably assess publication bias or small-study effects. The corresponding limit effect size was b = 0.72 (95% CI: −1.24 to 2.69), and the confidence interval also encompassed zero. Detailed results are presented in Figure 3B. Given the relatively small number of effect sizes and the dependency structure inherent in the three-level meta-analytic framework, these findings should be interpreted with caution. Overall, publication-bias assessments should be regarded as exploratory and interpreted cautiously due to the limited number of available effect sizes and the dependency structure inherent in the three-level meta-analytic framework.

Based on the GRADE evaluation, the overall certainty of evidence for knee muscle strength was rated as low. Although all included studies were randomized controlled trials, the certainty was downgraded due to concerns regarding risk of bias, inconsistency across intervention protocols and outcome measures, and imprecision resulting from the limited number of studies and wide confidence intervals.

The certainty of evidence for hop performance was rated as very low. The evidence was downgraded due to concerns regarding risk of bias, inconsistency, imprecision associated with the small sample size and limited number of studies, and uncertainty surrounding the pooled effect estimate.

### 3.3 Main effect results (knee muscle strength)

#### 3.3.1 Effects of strengthening exercise on knee muscle strength

**Figure 4:**
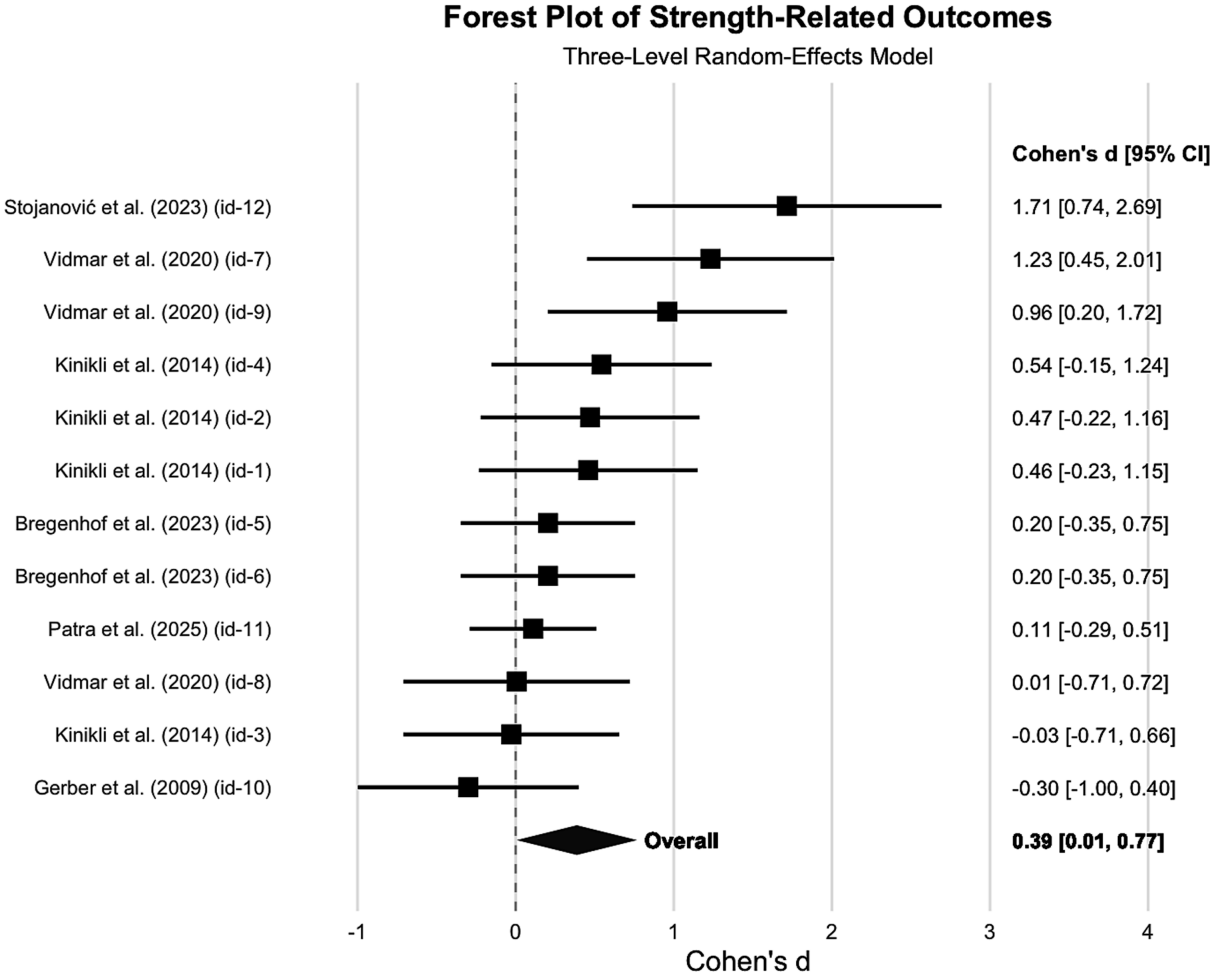
Forest plot of the pooled effects of strengthening interventions on knee muscle strength.

A three-level multivariate meta-analysis comprising 12 effect sizes demonstrated a statistically significant positive effect of strengthening interventions on knee muscle strength (*d* = 0.39, 95% CI 0.01–0.77, *p* = 0.046). Moderate heterogeneity was observed (*Q* = 22.67, *p* = 0.020; total *I*^2^ = 55.8%). Variance decomposition indicated that heterogeneity was attributable to both between-study (Level 3 *I*^2^ = 32.5%; τ^2^_Level 3 = 0.079) and within-study variability (Level 2 *I*^2^ = 23.3%; τ^2^_Level 2 = 0.057). Detailed results are presented in Figure 4.

#### 3.3.2 Effects of postoperative time as a moderator

**Figure 5:**
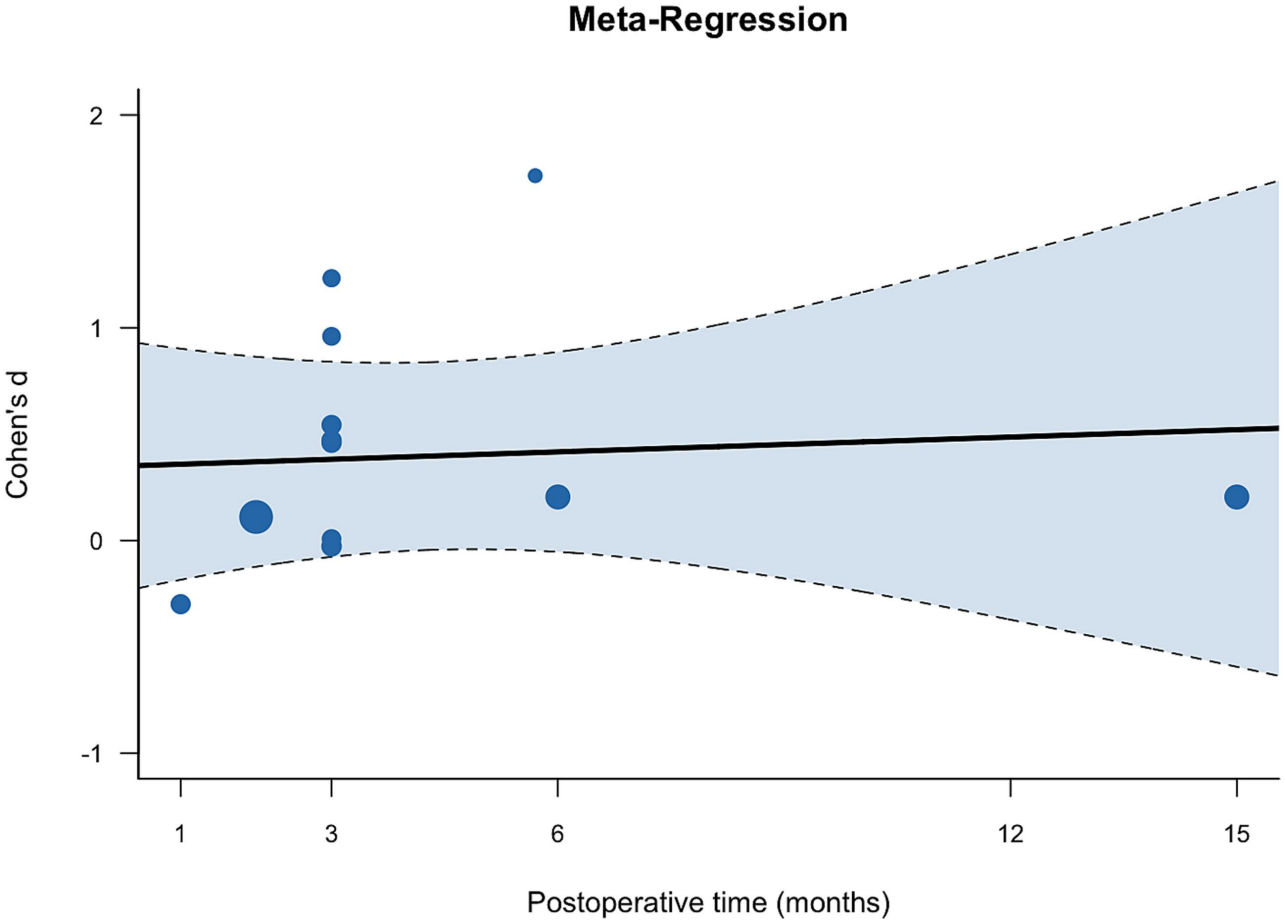
Relationship between postoperative time and the effects of strengthening interventions on strength-related outcomes after ACL reconstruction. Each circle represents an individual study effect size. Larger circles indicate greater study weight. The solid line shows the fitted meta-regression, and the shaded area indicates the 95% confidence interval.

The meta-regression incorporating postoperative time as a continuous moderator indicated no significant association with the effects of strengthening interventions on knee muscle strength (*F* = 0.073, *p* = 0.793). Specifically, postoperative time did not significantly predict variability in effect sizes (*k* = 12, *β* = 0.012, 95% CI − 0.084 to 0.107). Detailed results are shown in Figure 5.

#### 3.3.3 Effects of population type as a moderator

**Figure 6:**
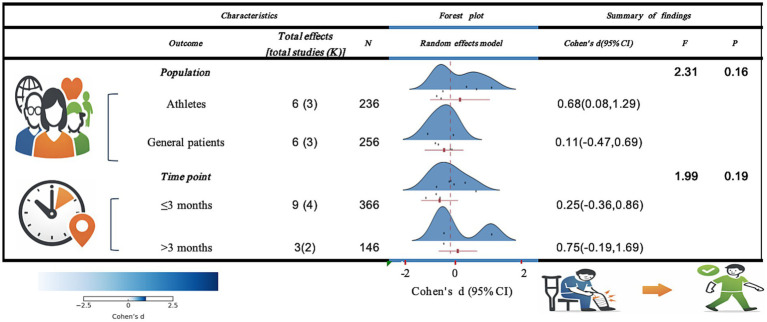
Moderator analysis of the effects of strengthening exercise on knee muscle strength stratified by population group (athletes vs. general patients) and time point (≤3 months vs. >3 months).

The meta-regression analysis indicated that population type did not significantly moderate the effects of strengthening exercise on knee muscle strength (*F* = 2.31, *p* = 0.159). Subgroup analyses showed that strengthening interventions produced a significant improvement in athletes (*d* = 0.68, 95% CI [0.08, 1.29], *p* = 0.031), whereas no significant effect was observed in general patients (*d* = 0.11, 95% CI [−0.47, 0.69], *p* = 0.677). Detailed results are presented in Figure 6.

#### 3.3.4 Effects of time point as a moderator

Subgroup analyses based on postoperative time point (≤3 months vs. >3 months) showed no significant moderating effect on the outcomes of knee muscle strength (*F* = 1.99, *p* = 0.188). The pooled effect size was *d* = 0.25 (95% CI [−0.36, 0.86], *p* = 0.380) for assessments conducted within 3 months postoperatively, and *d* = 0.75 (95% CI [−0.19, 1.69], *p* = 0.108) for assessments beyond 3 months. Detailed results are presented in Figure 6.

### 3.4 Main effect results (hop performance)

#### 3.4.1 Effects of strengthening exercise on hop performance

**Figure 7:**
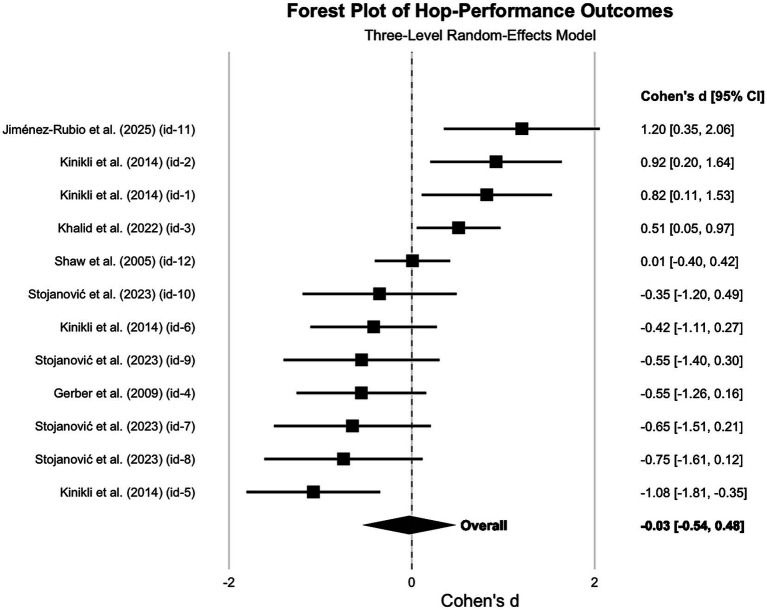
Forest plot of the pooled effects of strengthening interventions on hop performance.

A three-level multivariate meta-analysis comprising 12 effect sizes revealed no significant effect of strengthening interventions on hop performance (*d* = −0.03, 95% CI − 0.54 to 0.48, *p* = 0.906). Substantial heterogeneity was observed across effect sizes (*Q* = 43.33, *p* < 0.001; total *I*^2^ = 77.8%). Variance decomposition indicated that heterogeneity was primarily attributable to within-study variability (Level 2 *I*^2^ = 67.0%; τ^2^_Level 2 = 0.356), whereas between-study heterogeneity was minimal (Level 3 *I*^2^ = 10.8%; τ^2^_Level 3 = 0.058). Detailed results are presented in Figure 7.

#### 3.4.2 Effects of postoperative time as a moderator

**Figure 8:**
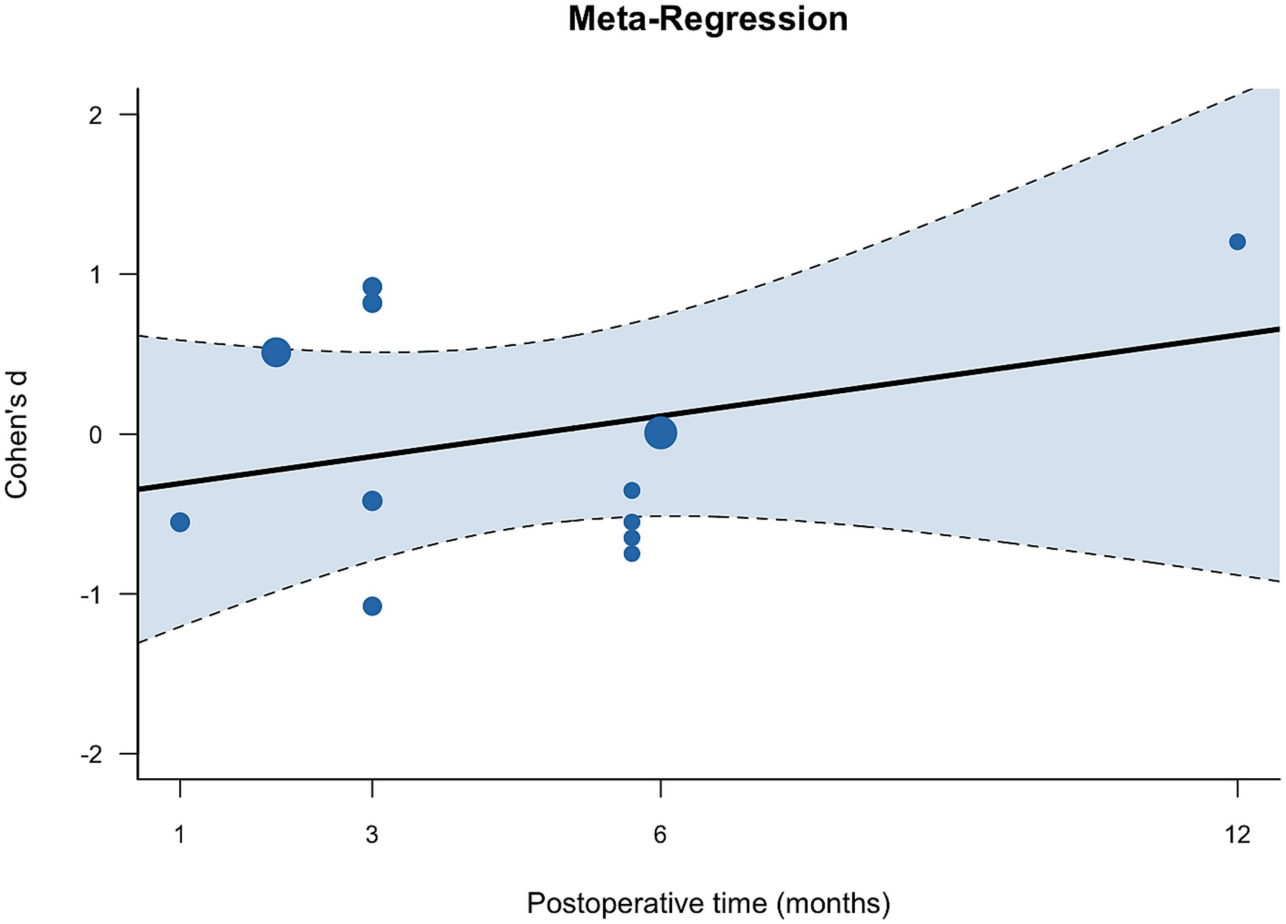
Relationship between postoperative time and the effects of strengthening interventions on hop performance after ACL reconstruction. Each circle represents an individual study effect size. Larger circles indicate greater study weight. The solid line shows the fitted meta-regression, and the shaded area indicates the 95% confidence interval.

The meta-regression incorporating postoperative time as a continuous moderator indicated no significant association with the effects of strengthening interventions on hop performance (*F* = 0.992, *p* = 0.343). Specifically, postoperative time did not significantly predict variability in effect sizes (*k* = 12, *β* = 0.084, 95% CI − 0.104 to 0.273, p = 0.343). Detailed results are presented in Figure 8.

#### 3.4.3 Effects of population type as a moderator

**Figure 9:**
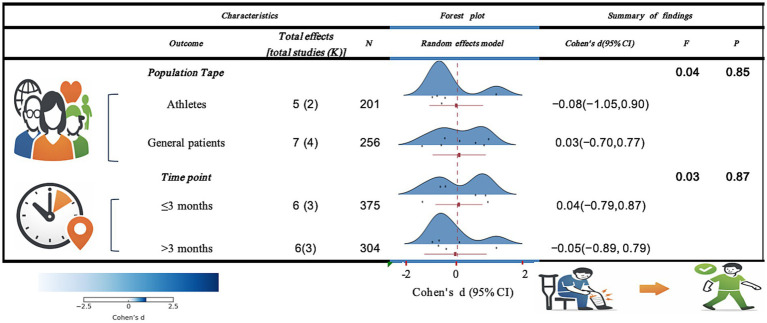
Moderator analysis of the effects of strengthening exercise on hop performance stratified by population group (athletes vs. general patients) and time point (≤3 months vs. >3 months).

The meta-regression analysis indicated that population type did not significantly moderate the effects of strengthening exercise on hop performance (*F* = 0.040, *p* = 0.846). Subgroup analyses showed no significant effects in either athletes (*d* = −0.08, 95% CI [−1.05, 0.90], *p* = 0.867) or general patients (*d* = 0.03, 95% CI [−0.70, 0.77], *p* = 0.921). Detailed results are presented in Figure 9.

#### 3.4.4 Effects of time point as a moderator

Subgroup analyses based on postoperative time point (≤3 months vs. >3 months) showed no significant moderating effect on hop performance outcomes (*F* = 0.03, p = 0.867). The pooled effect size was *d* = 0.04 (95% CI [−0.79, 0.87], *p* = 0.916) for assessments conducted within 3 months postoperatively, and *d* = −0.05 (95% CI [−0.89, 0.79], *p* = 0.896) for assessments beyond 3 months. Detailed results are presented in Figure 9.

## 4 Discussion

This three-level meta-analysis was conducted to examine the effects of strengthening exercise interventions on postoperative recovery following ACL reconstruction, with a particular focus on knee muscle strength and hop performance outcomes. The pooled results demonstrated that strength-oriented rehabilitation interventions were associated with improved knee muscle strength following ACL reconstruction. In contrast, no significant overall effect was observed for hop performance, suggesting that gains in muscle strength may not necessarily translate into improvements in functional hopping ability. Furthermore, meta-regression analyses revealed that postoperative time, population type, and assessment time point did not significantly moderate these outcomes. Collectively, these findings highlight that while strengthening interventions can enhance knee strength after ACL reconstruction, additional rehabilitation components may be required to optimize functional performance.

### 4.1 Effect of strengthening exercise on knee muscle strength

Our meta-analysis demonstrated that strengthening exercise interventions improved postoperative knee muscle strength following ACL reconstruction. The pooled effect indicated a moderate positive improvement in knee flexor strength, suggesting that resistance-based rehabilitation contributes meaningfully to muscular recovery after surgery. This finding is generally consistent with previous clinical evidence highlighting strengthening exercise as a cornerstone of ACL rehabilitation, given that persistent strength deficits are commonly reported even months after reconstruction (34). For example, a randomized trial of early progressive eccentric exercise after ACL reconstruction reported improvements in muscle size and functional outcomes compared with standard rehabilitation, supporting the potential benefits of progressive resistance-based approaches (28).

Similarly, a randomized controlled trial in athletes after ACL reconstruction found that structured isokinetic strength training improved knee muscle strength (and related sensorimotor outcomes), consistent with the premise that targeted strengthening can facilitate postoperative recovery (35). Potential mechanisms underlying these improvements may include muscle hypertrophy, enhanced motor unit recruitment, and recovery from postoperative neuromuscular inhibition. However, these mechanisms were not directly assessed in the included studies and therefore remain speculative. Taken together, the present findings suggest that strength-oriented rehabilitation interventions may contribute to improvements in knee muscle strength following ACL reconstruction. However, the observed effects should not be interpreted as evidence of uniform benefits across all postoperative stages, populations, or rehabilitation protocols.

### 4.2 Effect of strengthening exercise on hop performance

In contrast, our meta-analysis found that strengthening exercise did not produce a significant overall improvement in hop performance outcomes. This suggests that although strengthening interventions can enhance muscular strength, such gains may not directly translate into functional hopping ability, which is often considered a more complex indicator of dynamic knee stability and readiness for return to sport (4). This result is partly inconsistent with some individual studies in which plyometric- or power-oriented rehabilitation (often implemented alongside conventional strengthening) was associated with improved hop-related symmetry or functional performance during later-phase rehabilitation (36). However, it is also compatible with evidence indicating that return-to-sport outcomes are multifactorial and that hop/jump capacity is shaped by factors beyond isolated strength, including psychological readiness and broader rehabilitation exposure (37). Several mechanisms may explain this discrepancy. Hop performance depends not only on muscle strength but also on proprioception, movement coordination, psychological readiness, and sport-specific motor control, which may not be sufficiently addressed through strengthening alone.

Furthermore, the substantial heterogeneity observed across hop-related effect sizes suggests that differences in testing protocols and rehabilitation content may contribute to inconsistent functional responses; methodological work has also highlighted that hop test outcomes can be sensitive to test procedures and reporting practices (38). Overall, these findings indicate that while strengthening exercise is effective for restoring muscle capacity, additional task-specific and neuromuscular components may be required to optimize hop performance after ACL reconstruction.

### 4.3 Limitations and future directions

Several limitations should be considered when interpreting the present findings. First, the number of included studies and effect sizes was relatively small, which may have limited statistical power and increased uncertainty in the moderator analyses. A further limitation is the clinical heterogeneity of the included interventions and comparator conditions. Although all interventions were designed to enhance strength-related recovery after ACL reconstruction, they differed in training modality, loading characteristics, progression schemes, rehabilitation phase, and the nature of the comparison group. In several studies, strength-oriented rehabilitation protocols were compared with alternative rehabilitation approaches or different strength-oriented training strategies rather than a non-strengthening control condition. Owing to the limited number of studies, modality-specific subgroup analyses were not feasible. Therefore, the present findings should be interpreted as reflecting the overall effects of strength-oriented rehabilitation strategies following ACL reconstruction rather than the isolated effect of strengthening interventions compared with non-strengthening controls.

Another limitation of this review is that the pooled knee muscle strength outcome incorporated different strength assessment modalities, including quadriceps and hamstring strength measures as well as isometric, concentric, and eccentric testing protocols. Although these measures are commonly used to evaluate knee muscle strength following ACL reconstruction, they may reflect distinct physiological and functional characteristics. Due to the limited number of available studies, modality-specific subgroup analyses were not feasible. Therefore, the pooled estimate should be interpreted as representing overall knee muscle strength rather than any single strength component.

## 5 Conclusion

This three-level meta-analysis suggests that strength-oriented rehabilitation interventions can improve postoperative knee muscle strength following ACL reconstruction. However, no significant overall improvement was observed in hop-performance outcomes, indicating that gains in muscle strength may not necessarily translate into improvements in functional hopping performance. No statistically significant moderation effects were detected for postoperative time or population type. Overall, these findings support the potential role of strength-oriented rehabilitation interventions in postoperative recovery after ACL reconstruction, while highlighting the importance of rehabilitation strategies that address neuromuscular control, movement coordination, dynamic stability, and functional performance.

## Data Availability

The original contributions presented in the study are included in the article/Supplementary material, further inquiries can be directed to the corresponding author.

## References

[1] Bradsell H Frank RM. Anterior cruciate ligament injury prevention. Ann Joint. (2022) 7:1. doi: 10.21037/aoj-2020-01, 38529144 PMC10929369

[2] Ueki H Niga S Tsukada S Saito M Mori H Ikezawa Y . Acute anterior cruciate ligament injuries can be repaired through conservative treatment with early protective motion: a single-group cohort study. BMJ Open Sport Exerc Med. (2025) 11:e002802. doi: 10.1136/bmjsem-2025-002802, 41321646 PMC12658557

[3] Buckthorpe M Danelon F La Rosa G Nanni G Stride M Della Villa F. Recommendations for hamstring function recovery after ACL reconstruction. Sports Med. (2021) 51:607–24. doi: 10.1007/s40279-020-01400-x, 33332017

[4] Girdwood M Culvenor AG Rio EK Patterson BE Haberfield M Couch J . Tale of quadriceps and hamstring muscle strength after ACL reconstruction: a systematic review with longitudinal and multivariate meta-analysis. Br J Sports Med. (2025) 59:423–34. doi: 10.1136/bjsports-2023-107977, 39389762

[5] Sonnery-Cottet B Saithna A Quelard B Daggett M Borade A Ouanezar H . Arthrogenic muscle inhibition after ACL reconstruction: a scoping review of the efficacy of interventions. Br J Sports Med. (2019) 53:289–98. doi: 10.1136/bjsports-2017-098401, 30194224 PMC6579490

[6] McPherson AL Schilaty ND Anderson S Nagai T Bates NA. Arthrogenic muscle inhibition after anterior cruciate ligament injury: injured and uninjured limb recovery over time. Front Sports Active Living. (2023) 5:1143376. doi: 10.3389/fspor.2023.1143376, 37025459 PMC10072230

[7] West T Culvenor A Bruder A Crossley K. Hop tests predict future symptoms, function and return to sport after ACL injury: a systematic review and meta-analysis. J Sci Med Sport. (2021) 24:S61. doi: 10.1016/j.jsams.2021.09.154

[8] Girdwood MA Crossley KM Rio EK Patterson BE Haberfield MJ Couch JL . Hop to it! A systematic review and longitudinal Meta-analysis of hop performance after ACL reconstruction. Sports Med. (2025) 55:101–13. doi: 10.1007/s40279-024-02121-1, 39414723 PMC11787245

[9] Smiley T Dallman J Long R Kapple M Aldag L Mok A . Lower extremity return to sport testing: a systematic review. Knee. (2024) 50:115–46. doi: 10.1016/j.knee.2024.07.021, 39163752

[10] Welling W Benjaminse A Lemmink K Dingenen B Gokeler A. Progressive strength training restores quadriceps and hamstring muscle strength within 7 months after ACL reconstruction in amateur male soccer players. Phys Ther Sport. (2019) 40:10–8. doi: 10.1016/j.ptsp.2019.08.004, 31425918

[11] Baron JE Parker EA Duchman KR Westermann RW. Perioperative and postoperative factors influence quadriceps atrophy and strength after ACL reconstruction: a systematic review. Orthop J Sports Med. (2020) 8:2325967120930296. doi: 10.1177/2325967120930296, 32647734 PMC7328065

[12] Ambegaonkar JP Mettinger LM Caswell SV Burtt A Cortes N. Relationships between core endurance, hip strength, and balance in collegiate female athletes. Int J Sports Phys Ther. (2014) 9:604–16. 25328823 PMC4196325

[13] Augustsson J. Documentation of strength training for research purposes after ACL reconstruction. Knee Surg Sports Traumatol Arthrosc. (2013) 21:1849–55. doi: 10.1007/s00167-012-2167-3, 22898912

[14] Bafrouei MJ Khorramroo F Minoonejad H Mousavi SH. Strengthening exercises improve knee muscle strength and performance but not pain in ACL-reconstructed individuals: a systematic review and meta-analysis of randomised controlled trials. J Exp Orthop. (2025) 12:e70576. doi: 10.1002/jeo2.70576, 41416241 PMC12709656

[15] Cheung MW. Modeling dependent effect sizes with three-level meta-analyses: a structural equation modeling approach. Psychol Methods. (2014) 19:211–29. doi: 10.1037/a0032968, 23834422

[16] Kınıklı GI Yüksel I Baltacı G Atay OA. The effect of progressive eccentric and concentric training on functional performance after autogenous hamstring anterior cruciate ligament reconstruction: a randomized controlled study. Acta Orthop Traumatol Turc. (2014) 48:283–9. doi: 10.3944/AOTT.2014.13.0111, 24901918

[17] Vidmar MF Baroni BM Michelin AF Mezzomo M Lugokenski R Pimentel GL . Isokinetic eccentric training is more effective than constant load eccentric training for quadriceps rehabilitation following anterior cruciate ligament reconstruction: a randomized controlled trial. Braz J Phys Ther. (2020) 24:424–32. doi: 10.1016/j.bjpt.2019.07.003, 31351901 PMC7563799

[18] Van den Noortgate W López-López JA Marín-Martínez F Sánchez-Meca J. Three-level meta-analysis of dependent effect sizes. Behav Res Methods. (2013) 45:576–94. doi: 10.3758/s13428-012-0261-6 23055166

[19] Assink M Wibbelink C. Fitting three-level meta-analytic models in R: a step-by-step tutorial. Quant Methods Psychol. (2016) 12:154–74. doi: 10.20982/tqmp.12.3.p154

[20] Page MJ McKenzie JE Bossuyt PM Boutron I Hoffmann TC Mulrow CD . The PRISMA 2020 statement: an updated guideline for reporting systematic reviews. BMJ. 372:n71. doi: 10.1136/bmj.n71, 33782057 PMC8005924

[21] Sterne JAC Savović J Page MJ Elbers RG Blencowe NS Boutron I . RoB 2: a revised tool for assessing risk of bias in randomised trials. BMJ. (2019) 366:l4898. doi: 10.1136/bmj.l4898, 31462531

[22] Egger M Davey Smith G Schneider M Minder C. Bias in meta-analysis detected by a simple, graphical test. BMJ. (1997) 315:629–34. doi: 10.1136/bmj.315.7109.629, 9310563 PMC2127453

[23] Sterne JA Sutton AJ Ioannidis JP Terrin N Jones DR Lau J . Recommendations for examining and interpreting funnel plot asymmetry in meta-analyses of randomised controlled trials. BMJ. (2011) 343:d4002–2. doi: 10.1136/bmj.d4002 21784880

[24] Guyatt GH Oxman AD Vist GE Kunz R Falck-Ytter Y . GRADE: an emerging consensus on rating quality of evidence and strength of recommendations. BMJ. (2008) 336:924–6. doi: 10.1136/bmj.39489.470347.AD, 18436948 PMC2335261

[25] Higgins JP Thompson SG. Quantifying heterogeneity in a meta-analysis. Stat Med. (2002) 21:1539–58. doi: 10.1002/sim.1186, 12111919

[26] Viechtbauer W. Conducting meta-analyses in R with the metafor package. J Stat Softw. (2010) 36:1–48. doi: 10.18637/jss.v036.i03

[27] Bregenhof B Aagaard P Nissen N Creaby MW Thorlund JB Jensen C . The effect of progressive resistance exercise on knee muscle strength and function in participants with persistent hamstring deficit following ACL reconstruction: a randomized controlled trial. J Orthop Sports Phys Ther. (2023) 53:40–8. doi: 10.2519/jospt.2022.11360, 36306171

[28] Gerber JP Marcus RL Dibble LE Greis PE Burks RT LaStayo PC. Effects of early progressive eccentric exercise on muscle size and function after anterior cruciate ligament reconstruction: a 1-year follow-up study of a randomized clinical trial. Phys Ther. (2009) 89:51–9. doi: 10.2522/ptj.20070189, 18988664

[29] Patra RC Gupta S Yashudas A Mahajan S. Effects of isoinertial training on muscle power, endurance, isometric strength, and balance: randomized clinical trial in patients with post-ACL reconstruction. Trials. (2025) 26:257. doi: 10.1186/s13063-025-08953-0, 40713700 PMC12297821

[30] Stojanović MDM Andrić N Mikić M Vukosav N Vukosav B Zolog-Șchiopea DN . Effects of eccentric-oriented strength training on return to sport criteria in late-stage anterior cruciate ligament (ACL)-reconstructed professional team sport players. Medicina (Kaunas). (2023) 59:1111. doi: 10.3390/medicina59061111, 37374316 PMC10305302

[31] Khalid K Anwar N Saqulain G Afzal MF. Neuromuscular training following anterior cruciate ligament reconstruction - pain, function, strength, Power & Quality of life perspective: a randomized control trial. Pakis J Med Sci. (2022) 38:2175–81. doi: 10.12669/pjms.38.8.5730, 36415269 PMC9676616

[32] Jiménez-Rubio S García-Calvo T Martínez-Aranda LM Raya-González J. A specific reconditioning training program implemented 12 months after ACL surgery improves lower-limb jump variables in amateur soccer players. Front Physiol. (2025) 16:1630156. doi: 10.3389/fphys.2025.1630156, 40895437 PMC12392115

[33] Shaw T Williams MT Chipchase LS. Do early quadriceps exercises affect the outcome of ACL reconstruction? A randomised controlled trial. Aust J Physiother. (2005) 51:9–17. doi: 10.1016/s0004-9514(05)70048-9, 15748120

[34] Brown C Marinko L LaValley MP Kumar D. Quadriceps strength after anterior cruciate ligament reconstruction compared with uninjured matched controls: a systematic review and Meta-analysis. Orthop J Sports Med. (2021) 9:2325967121991534. doi: 10.1177/2325967121991534, 33889639 PMC8040575

[35] Wang K Cheng L Wang B He B. Effect of isokinetic muscle strength training on knee muscle strength, proprioception, and balance ability in athletes with anterior cruciate ligament reconstruction: a randomised control trial. Front Physiol. (2023) 14:1237497. doi: 10.3389/fphys.2023.1237497, 37795267 PMC10546193

[36] Kasmi S Zouhal H Hammami R Clark CCT Hackney AC Hammami A . The effects of eccentric and plyometric training programs and their combination on stability and the functional performance in the post-ACL-surgical rehabilitation period of elite female athletes. Front Physiol. (2021) 12:688385. doi: 10.3389/fphys.2021.688385, 34276409 PMC8283277

[37] Niederer D Keller M Jakob S Wießmeier M Petersen W Schüttler KF . Rehabilitation volume, psychological readiness, and motor function are important factors for a successful return to sport after anterior cruciate ligament reconstruction: a 2-year follow-up cohort study. J Sci Med Sport. (2025) 28:553–62. doi: 10.1016/j.jsams.2025.02.010, 40089432

[38] Read P Mc Auliffe S Wilson MG Myer GD. Better reporting standards are needed to enhance the quality of hop testing in the setting of ACL return to sport decisions: a narrative review. Br J Sports Med. (2021) 55:23–9. doi: 10.1136/bjsports-2019-101245, 32522734 PMC7788201

[39] R Core Team. R: A language and environment for statistical computing. Vienna, Austria: R Foundation for Statistical Computing (2023). Available online at: https://www.R-project.org/

