# Supplementary material for: Effectiveness of strength-oriented rehabilitation interventions as a non-pharmacologic rehabilitation strategy for knee function after anterior cruciate ligament reconstruction: a three-level meta-analysis
Source: Front Med (Lausanne). 2026 Jun 24;13:1871508. doi: 10.3389/fmed.2026.1871508 (PMC13341512; doi:10.3389/fmed.2026.1871508)
Supplement: Supplementary file 1 [file Data_Sheet_1.DOCX]

**Supplementary File 1: search strategy**

| Database | Retrieval strategy |
| --- | --- |
| Cochrane | ((MeSH descriptor: [Anterior Cruciate Ligament Reconstruction] explode all trees OR "anterior cruciate ligament reconstruction":ti,ab,kw OR "ACL reconstruction":ti,ab,kw OR ACLR:ti,ab,kw OR "ACL-R":ti,ab,kw OR "ACL surgery":ti,ab,kw) AND (MeSH descriptor: [Resistance Training] explode all trees OR "strength training":ti,ab,kw OR "resistance training":ti,ab,kw OR "strength exercise*":ti,ab,kw OR "eccentric training":ti,ab,kw OR "concentric training":ti,ab,kw OR "isometric training":ti,ab,kw OR "neuromuscular training":ti,ab,kw OR "quadriceps strengthening":ti,ab,kw) AND (MeSH descriptor: [Muscle Strength] explode all trees OR strength*:ti,ab,kw OR "muscle strength":ti,ab,kw OR power:ti,ab,kw OR performance:ti,ab,kw OR function*:ti,ab,kw OR "hop test*":ti,ab,kw OR "single-leg hop":ti,ab,kw OR jump*:ti,ab,kw OR "jumping performance":ti,ab,kw)) |
| Embase | (('anterior cruciate ligament reconstruction'/exp OR 'anterior cruciate ligament reconstruction':ti,ab,kw OR 'ACL reconstruction':ti,ab,kw OR ACLR:ti,ab,kw OR 'ACL-R':ti,ab,kw OR 'ACL surgery':ti,ab,kw) AND ('resistance training'/exp OR 'strength training':ti,ab,kw OR 'resistance training':ti,ab,kw OR 'strength exercise*':ti,ab,kw OR 'eccentric training':ti,ab,kw OR 'concentric training':ti,ab,kw OR 'isometric training':ti,ab,kw OR 'neuromuscular training':ti,ab,kw OR 'quadriceps strengthening':ti,ab,kw) AND ('muscle strength'/exp OR strength*:ti,ab,kw OR 'muscle strength':ti,ab,kw OR power:ti,ab,kw OR performance:ti,ab,kw OR function*:ti,ab,kw OR 'hop test*':ti,ab,kw OR 'single-leg hop':ti,ab,kw OR jump*:ti,ab,kw OR 'jumping performance':ti,ab,kw)) |
| Web of Science | TS=(("anterior cruciate ligament reconstruction" OR "ACL reconstruction" OR ACLR OR "ACL-R" OR "ACL surgery") AND ("strength* training" OR "resistance training" OR "strength* exercise*" OR "eccentric training" OR "concentric training" OR "isometric training*" OR "neuromuscular training" OR "quadriceps strengthening") AND (strength* OR "muscle strength" OR power OR performance OR function* OR "hop test*" OR "single-leg hop" OR jump* OR "jumping performance")) |
| PubMed | (("Anterior Cruciate Ligament Reconstruction"[Mesh] OR "anterior cruciate ligament reconstruction"[tiab] OR "ACL reconstruction"[tiab] OR ACLR[tiab] OR "ACL-R"[tiab] OR "ACL surgery"[tiab]) AND ("Resistance Training"[Mesh] OR "strength training"[tiab] OR "resistance training"[tiab] OR "strength exercise*"[tiab] OR "eccentric training"[tiab] OR "concentric training"[tiab] OR "isometric training"[tiab] OR "neuromuscular training"[tiab] OR "quadriceps strengthening"[tiab]) AND ("Muscle Strength"[Mesh] OR strength*[tiab] OR "muscle strength"[tiab] OR power[tiab] OR performance[tiab] OR function*[tiab] OR "hop test*"[tiab] OR "single-leg hop"[tiab] OR jump*[tiab] OR "jumping performance"[tiab])) |
| SPORTDiscus (via EBSCOhost) | ((MH "Anterior Cruciate Ligament Reconstruction" OR TX "anterior cruciate ligament reconstruction" OR TX "ACL reconstruction" OR TX ACLR OR TX "ACL-R" OR TX "ACL surgery") AND (MH "Resistance Training" OR TX "strength training" OR TX "resistance training" OR TX "strength exercise*" OR TX "eccentric training" OR TX "concentric training" OR TX "isometric training" OR TX "neuromuscular training" OR TX "quadriceps strengthening") AND (MH "Muscle Strength" OR TX strength* OR TX "muscle strength" OR TX power OR TX performance OR TX function* OR TX "hop test*" OR TX "single-leg hop" OR TX jump* OR TX "jumping performance")) |
